# Spatial and Proteolytic Determinants of Therapeutic Potential in HER2‐Low Breast Cancer

**DOI:** 10.1002/advs.74975

**Published:** 2026-03-24

**Authors:** Deqian Xie, Wei Dai, Guangzhen Wu

**Affiliations:** ^1^ Department of Urology The First Affiliated Hospital of Dalian Medical University Dalian Liaoning China; ^2^ Operating Room The First Affiliated Hospital of Dalian Medical University Dalian Liaoning China

To the Editor:

We read with great interest the outstanding work by Xu et al. recently published in Advanced Science, “Proteogenomic Characterization Reveals Subtype‐Specific Therapeutic Potential for HER2‐Low Breast Cancer.” Through high‐throughput integrative proteomics and genomics analysis, the authors meticulously mapped the molecular landscape of HER2‐low breast cancer and identified molecular clusters with specific therapeutic vulnerabilities [1]. This work provides a valuable data resource for understanding the emerging and complex clinical entity of HER2‐low expression. However, integrating recent breakthroughs in the pharmacology of antibody‐drug conjugates and spatial biology, we aim to offer complementary perspectives and innovative insights into the biological essence of HER2‐low tumors and the mechanisms determining their therapeutic potential. We propose that the therapeutic potential of HER2‐low tumors, particularly in the context of novel ADCs like Trastuzumab Deruxtecan, may be determined not solely by intracellular signaling subtypes, but critically by the spatial topology of the tumor microenvironment and proteolytic competence.

Evidence from large‐scale genomic analyses indicates that HER2‐low breast cancer is not a distinct molecular entity defined by unique driver mutations [2]. After adjusting for hormone receptor status, HER2‐low tumors exhibit high homogeneity with HER2‐zero tumors in genomic mutation profiles, including PIK3CA and TP53 mutation frequencies and transcriptomic characteristics like the PAM50 classification [2, 3]. The subtype‐specific proteomic features observed by the original authors may reflect continuous changes in estrogen receptor signaling intensity or metabolic adaptations within a luminal biological context, rather than unique oncogenic programs directly driven by low HER2 expression [4]. Viewing HER2 low expression as a phenotypic continuum spanning diverse molecular backgrounds, rather than a discrete intrinsic subtype, appears biologically more accurate and clinically more instructive. This understanding helps explain why novel ADCs like T‐DXd demonstrate therapeutic efficacy beyond traditional subtype boundaries, extending to ultra‐low HER2 expression populations. Based on this genome‐based phenotypic continuum, the research focus should transition from intracellular signaling toward pharmacological intervention mechanisms within the extracellular microenvironment.

This study primarily focuses on the proteomic characteristics within tumor cells, which may present limitations when explaining the efficacy of next‐generation ADCs like Trastuzumab Deruxtecan. Traditional ADC pharmacology emphasizes receptor‐mediated endocytosis and lysosomal cleavage; however, in HER2‐low tumors, receptor density is often insufficient to support lethal levels of efficient endocytosis [5]. Recent research reveals a disruptive mechanism: extracellular cathepsin L, abundant in the tumor microenvironment, can directly cleave the linker of T‐DXd extracellularly, releasing a membrane‐permeable carrier that achieves tumor cell killing [6]. This implies that the key biomarker determining the “therapeutic potential” of HER2‐low tumors may extend beyond intracellular signaling protein abundance to encompass the proteolytic competence of the extracellular protease environment. To this end, we encourage the authors to re‐mine their existing proteomics datasets to evaluate the abundance of CTSL and related matrix metalloproteinases. Correlating these extracellular proteases with the proposed treatment response clusters and subsequently integrating secretome data in future studies could uncover biomarkers with greater predictive value than intracellular signaling pathways alone.

Bulk proteomics inevitably obscures spatial heterogeneity within tumors, where HER2‐low tumors often exhibit mosaic patterns of HER2‐positive and ‐negative clones [7]. While the renowned bystander effect of ADCs can overcome some heterogeneity, its efficacy remains limited by physical distance and matrix barriers. Research indicates that the spatial topology of ERBB2 expression directly determines antibody binding efficiency and carrier diffusion radius [8]. In regions with extremely low HER2 expression, drugs struggle to reach targets even when proteomics indicate pathway activation. This limitation arises either from insufficient antigen anchors for drug enrichment or from matrix barriers impeding carrier diffusion [9]. Future studies should integrate spatial transcriptomics or spatial proteomics technologies to incorporate HER2 spatial clustering and the physical permeability of the matrix. Considering the high costs associated with spatial multi‐omics, AI‐based digital pathology could serve as a more clinically feasible auxiliary tool for extracting HER2 spatial clustering features, facilitating the practical application of these concepts, and improving the predictive efficacy of molecular subtyping.

The release of DESTINY‐Breast06 trial results has extended the beneficiary population for T‐DXd to include patients with HER2 ultra‐low expression, defined as IHC greater than 0 but less than 1+. This finding blurs the distinction between HER2‐low and HER2‐zero expression [10]. If the HER2‐zero control group in this study included a proportion of ultra‐low expression samples that remain biologically active at the molecular level despite negative IHC detection, genuine biological differences may be obscured. Future proteomic characterization should incorporate this ultra‐low expression subgroup by utilizing high‐sensitivity mass spectrometry to redefine the baseline for HER2‐negative status. Within this ultra‐low expression cohort, where antigen anchors are scarce, the spatial topology of the microenvironment and the cleavage capacity of extracellular proteases become highly relevant. Such an approach prevents overlooking potential biological signals falling below the threshold of conventional immunohistochemical detection and eliminates the inherent inter‐observer variability associated with traditional IHC.

We congratulate the authors on this impressive work. Introducing the concept of a spatial‐proteolytic‐phenotypic continuum that integrates static omics data with dynamic drug release mechanisms and the spatial microenvironment will enable a more accurate definition of the therapeutic potential of this cohort. This approach facilitates the transition from biological subtyping to pharmacological stratification.

## Conflicts of Interest

The authors declare no conflicts of interest.

## Data Availability

Data sharing not applicable to this article as no datasets were generated or analysed during the current study.
